# Knowledge change regarding osteoporosis prevention: translating recommended guidelines into user-friendly messages within a community forum

**DOI:** 10.1186/s13104-015-0985-3

**Published:** 2015-02-08

**Authors:** Sarah M Hosking, Amelia G Dobbins, Julie A Pasco, Sharon L Brennan

**Affiliations:** Epi-Centre for Healthy Ageing, School of Medicine (Barwon Health), Deakin University, PO BOX 281, Geelong, VIC Australia; NorthWest Academic Centre, Department of Medicine, the University of Melbourne, St Albans, VIC Australia

**Keywords:** Osteoporosis, Knowledge change, Recommended guidelines, Prevention

## Abstract

**Background:**

Osteoporosis is a skeletal disorder characterised by low bone mineral density and increased fracture risk. Nationally the total costs of this chronic disease are currently estimated at $2.754 billion annually. Effective public health messages providing clear recommendations are vital in supporting prevention efforts. This research aimed to investigate knowledge change associated with the translation of preventive guidelines into accessible messages for the community.

**Findings:**

We delivered a community-based information session that translated recommended guidelines for osteoporosis prevention into lay terms; items focused on dietary calcium, vitamin D, physical activity, alcohol, smoking and general osteoporosis-related knowledge. We developed a 10-item questionnaire reflecting these key points (score range 0–10) and investigated knowledge change associated with the session. Pre- and post-test questionnaires were completed by 47 participants (51% female), aged 21–94 years. Relatively high pre-test scores were observed for questions regarding sedentary activity and calcium intake. The lowest pre-test scores were observed for the item concerning whether swimming and cycling strengthened bones, and the highest possible score post-test was achieved for three of the items: calcium-rich food as a protective factor, and excessive alcohol and smoking as risk factors. The overall increase in knowledge change was a mean score of +2.08 (95%CI 1.58–2.42).

**Conclusions:**

An increase in knowledge regarding osteoporosis prevention was demonstrated over the short-term. Our findings suggest that the guidelines concerning dietary calcium are generally well understood; however, the asymptomatic nature of osteoporosis and the types of physical activity that assist with bone strength are less well understood.

## Background

Osteoporosis is a skeletal disorder characterised by low bone mineral density (BMD), microarchitectural deterioration of the bone and a subsequent increase in fracture risk [1]. Recent Australian data suggest that 330,000 women and 80,000 men have osteoporosis [2], and in the Geelong region specifically prevalence of osteoporosis has been estimated as 5.9% for men and 22.8% for women over the age of 50 [3]. Nationally, the total direct and indirect costs of this chronic disease are currently estimated at $2.754 billion [2], and set to increase dramatically in light of our ageing population. Effective public health messages that provide clear recommendations and develop osteoporosis-related knowledge are vital in supporting efforts in osteoporosis prevention. Although the modification of lifestyle behaviours may contribute significantly to reducing the risk of this disease, Australian communities have been reported in a number of studies as having a lack of knowledge about osteoporosis [4-7], and thus a limited ability to competently reduce their risk of this disease by modifying their lifestyles.

In efforts to address the increasing prevalence of osteoporosis, Osteoporosis Australia (OA) recently published an evidence-informed strategy for the prevention of this disease in the form of a consistent set of recommended guidelines [8]. The challenge is translating these comprehensive and evidence-based guidelines for a wider audience to ensure the messages reach beyond health professionals and are heeded by individuals who are at greatest risk of the disease.

World Osteoporosis Day was established by the International Osteoporosis Foundation in 1996, and is marked each year on October 20th by a variety of events in different countries aimed at raising awareness about the prevention, diagnosis and treatment of osteoporosis. We used this annual event as a platform to facilitate the translation of OA guidelines via an information session for the general community using visual and verbal tools. We aimed to investigate short term knowledge change associated with the translation of recommended preventive guidelines to accessible messages for the general community.

### Hypothesis

We anticipated that the recommended guidelines translated into user-friendly messages presented in the form of a community-based information session, would increase participants’ knowledge of prevention messages.

## Methods

### Participants

Participants were recruited to attend a World Osteoporosis Day event in October 2013 using a convenience sample method. We delivered a community-based information session that translated the recommended guidelines for osteoporosis prevention; guidelines focused on dietary calcium, vitamin D exposure, physical activity, alcohol consumption, smoking and general understanding of osteoporosis. The event was open to the public and advertised broadly in the local newspaper as of interest to all adults, and fliers for the event were sent to participants (aged ≥50 years) enrolled in the Geelong Osteoporosis Study (GOS), a cohort randomly recruited from the Barwon Statistical Division (BSD), south eastern Australia [9]. Attendees at previous GOS public forums were also invited by mail to attend, and fliers were distributed to community groups in the BSD who assisted in the creation of an oversized jigsaw puzzle that was used as a communication tool during the information session [10]. Ethics approval was provided by Barwon Health Human Research and Ethics Committee; completion of the questionnaire was taken as implied consent for participation and aligned with ethical approval.

### Outcome measure

We developed a 10-item questionnaire (see Table 1) that addressed the OA recommended prevention guidelines concerning osteoporosis [8]. Participants were asked to complete the questionnaire immediately prior to the information session, and again directly after the conclusion of the 40 minute information session. Participants had three possible response options for each of the 10 items on the questionnaire: True, False or Unsure. Research staff provided on-site assistance for vision impaired attendees who were unable to complete the questionnaire unaided; during the post-test, the assisting research staff members were blinded to pre-test responses. For analyses the questionnaire responses were coded as 1 if answered correctly, while incorrect and unsure responses were coded as 0.

**Table 1:** **10-item questionnaire addressing the OA recommended prevention guidelines concerning osteoporosis**

**Please read the following statements, and circle whether you think the statements are TRUE or FALSE. If you do not know the answer, please circle UNSURE**			
A diet low in calcium increases the risk of osteoporosis	TRUE	FALSE	USURE
We should include 3–5 serves of calcium-rich foods in our daily diet	TRUE	FALSE	USURE
Food is the main source of vitamin D	TRUE	FALSE	USURE
The body needs vitamin D to help absorb calcium	TRUE	FALSE	USURE
Excessive alcohol is bad for your bones	TRUE	FALSE	USURE
Activities like swimming and cycling help to build strong bones	TRUE	FALSE	USURE
People with osteoporosis can feel their bones getting weak	TRUE	FALSE	USURE
Long periods of sitting are good for bone strength	TRUE	FALSE	USURE
Cigarette smoking will harm your bones	TRUE	FALSE	USURE
Osteoporosis can affect men	TRUE	FALSE	USURE

### Statistical analyses

Of the 48 attendees at the event, all but one had completed both the pre- and post-test questionnaire; thus, after excluding this individual, our sample included 47 participants.

The mean change in overall pre- and post-test scores was determined using a paired t-test. Paired t-tests were also performed to detect changes in mean scores for each item. Two of the 47 participants had missed answering one question, one at pre-test and one at post-test; to account for these missing data we applied a conservative approach consistent with the null hypothesis that no knowledge change would be achieved and carried the last value forward or backward for each of these individuals as appropriate. We also performed a sensitivity analysis after excluding the two participants who had each missed answering one item (n = 45). Significance was set at *p*-value ≤0.05, and analyses were performed using Minitab (Version 16; Minitab, State College, PA, USA).

## Results

Ages of the 47 participants (51% female) who answered both the pre- and post-questionnaire ranged from 21–94 years (median 60 years). The pre-test knowledge of our participants was a combined score of 336 out of a possible 470 (71.5%) *vs.* the post-test score of 430 (91.5%).

**Figure 1:**
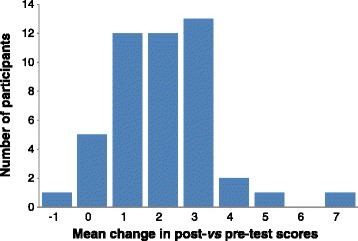
**Frequency of mean change in post-** ***vs*** **pre-test scores (all 10 questionnaire items combined) among 47 participants.**

Table 2 presents the pre- and post-test scores for each of the 10 individual item themes together with the mean change in scores. The highest pre-test scores were observed for the following items: low dietary calcium as a risk factor (0.91, 95%CI 0.83, 1.00), 3–5 serves of calcium-rich food as a protective factor (0.94, 95%CI 0.86, 1.01), and long periods of sitting as a risk factor for osteoporosis (0.92, 95%CI 0.83, 1.00). The lowest pre-test scores were observed for the item that questioned whether swimming and cycling strengthened bones, for which the pre-test score was 0.06 (95%CI-0.10, 0.14). However, it was this latter item that showed the greatest mean increase of 0.70 (95%CI 0.57, 0.84). The highest possible score post-test was achieved by participants for three of the items: 3–5 serves of calcium-rich food as a protective factor, and excessive alcohol and smoking as risk factors. The overall mean change in knowledge scores was +2.08 (95%CI 1.58, 2.42). Our sensitivity analysis showed a similar mean increase in scores. Figure 1 presents the spread of participants showing a difference in total scores from pre- to post-test; the majority of participants increased their knowledge by between 1–3 points.

**Table 2:** **Mean scores (95%CI) for each of the ten questions; significant results are in boldface**

	**Mean score (95%CI)**		
**Question Theme**	**Pre-test**	**Post-test**	**Change**
Diet low in calcium is a risk factor	0.91 (0.83, 1.00)	0.94 (0.86, 1.01)	+0.02 (−0.09, 0.14)
3–5 serves per day of calcium-rich foods are recommended	0.94 (0.86, 1.01)	1.00 (1.00, 1.00)	+0.06 (−0.09, 0.14)
Sunlight is the main source of vitamin D	0.60 (0.45, 0.74)*	0.77 (0.64, 0.89)	**+0.17 (0.02, 0.32)**
Body needs vitamin D to help absorb calcium	0.79 (0.67, 0.91)	0.98 (0.64, 0.89)*	**+0.19 (0.06, 0.32)**
Excessive alcohol is a risk factor	0.79 (0.67, 0.91)	1.00 (1.00, 1.00)	**+0.21 (0.09, 0.33)**
Swimming and cycling do not build bone strength	0.06 (−0.01, 0.14)	0.77 (0.64, 0.89)	**+0.70 (0.57, 0.84)**
Osteoporosis is asymptomatic	0.47 (0.32, 0.62)	0.83 (0.72, 0.94)	**+0.36 (0.22, 0.50)**
Long periods of sitting are a risk factor	0.92 (0.83, 1.00)	0.92 (0.83, 1.00)	+0.00 (−0.12, 0.12)
Smoking is a risk factor	0.79 (0.67, 0.91)	1.00 (1.00, 1.00)	**+0.21 (0.09, 0.33)**
Osteoporosis affects men	0.89 (0.80, 0.99)	0.96 (0.90, 1.02)	+0.06 (−0.01, 0.14)
Change in total score (all items combined)			**2.08 (1.57, 2.42)**

*Missing data: n = 1 for each question at the time point indicated.

## Discussion

We showed an increase in knowledge regarding osteoporosis prevention with pre- and post-tests performed immediately before and after the information session. This suggests that providing easily accessible messages to the general community can positively influence knowledge change regarding osteoporosis prevention. Our pre-test scores showed that, in general, recommended guidelines concerning dietary calcium intake are well understood; however, the asymptomatic nature of osteoporosis and the types of physical activity that strengthen bones are less well understood.

It is clear that the lifestyle behaviours involving dietary calcium, vitamin D exposure, physical activity, smoking and alcohol consumption influence the risk of osteoporosis [8], and given that these factors are all modifiable, they are often the prime focus of public health messages. It is likely due to awareness-raising activities in the arenas of public health, media and primary and secondary care, that we observed high pre-test scores for questions concerning adequate dietary calcium and vitamin D levels. Similarly, participants had prior understanding that smoking and alcohol were risk factors for poor bone health and achieved a 100% score post-test. In light of the well- documented links between many other chronic diseases and alcohol use and smoking, it is plausible that participants readily accepted from our information session that this link also exists for osteoporosis. Finally, the pre-test scores regarding physical activity indicated that while most participants understood that sedentary activity was a risk factor for osteoporosis, very few understood the specific types of activities that improved bone strength. Despite this latter item showing the greatest increase in knowledge change, it nevertheless remained one of the two lowest scoring items post-test. The lack of understanding regarding types of physical activity beneficial for bones may stem from the fact that the guidelines for osteoporosis prevention differ from the guidelines for general well-being where aerobic physical activity is considered positive for health [11], while weight bearing activities are useful for strengthening bones [8].

The pre- and post-test scores indicated that participants had a reasonably sound understanding that osteoporosis affects both sexes. In contrast, prior to the information session, less than half of the participants were aware that osteoporosis is often asymptomatic prior to a fracture occurring. It is of public health concern that, despite much research and media attention, many individuals remain unaware of the asymptomatic nature of osteoporosis and thus plausibly will disregard the need for preventive behaviours or health-related advice in the absence of symptoms. The limited community awareness regarding osteoporosis being asymptomatic pre-fracture has previously been reported, for instance Francis et al. [12] in 2009 and Solomon et al. in 2006 [13]; clearly, our targeted efforts during the last few years to raise awareness about osteoporosis being the ‘silent disease’ need to continue on a broad scale. It is imperative that public health remains focused on osteoporosis prevention *per se* rather than only directed toward those who have already fractured. Given that deficits in osteoporosis-related knowledge have also been reported in general practice [14,15], influencing a shift in the community’s understanding of osteoporosis will assist in sharing the role of osteoporosis prevention between multiple players.

One of the strengths of this study was that our information session and the 10-item questionnaire were developed to reflect the most recent OA guidelines [8]. Whilst we employed a convenience sampling method for recruitment, we aimed to include a larger age range of participants by specifically targeting the invitations to increase attendance by older individuals from across the BSD. Our information session resulted in a significant increase in knowledge change for 87% of the participants. However, we also acknowledge that five individuals did not show an improvement and one participant showed poorer knowledge following the information session; we speculate that this may be explained by poorer cognitive functioning and/or hearing in some of our older participants. This study also has some limitations. Due to the small sample size involved in this study we acknowledge that our findings might not be generalisable to other groups or populations; however, we are unable to comment further on this as no data pertaining to osteoporosis status or demographics were obtained. It is also possible that the differences we detected in knowledge change may be due to the participation bias inherent in a self-selected sample such as ours. We speculate that attendees who chose to be involved may have had different levels of knowledge prior to the information session and/or different post-test scores compared to the general population, due to a potential higher level of interest in the topic and a willingness to learn. The short time between the information session and the post-test questionnaires meant that only short-term knowledge change could be examined and thus we cannot speculate on the longer term benefit of the forum. However, previous studies have shown that even single sessions can result in a sustained improvement in knowledge [16]. We also acknowledge that increased knowledge is but one component of intervention strategies that are important for influencing behavioural change [17]. Finally, the 10-item questionnaire used to examine knowledge change was developed by researchers for this specific purpose, and has not been validated. Nevertheless, it is important to stress that the questions were based on current recommended guidelines.

## Conclusion

In conclusion, we focused our information session on translating into lay terms the currently-recommended guidelines regarding osteoporosis prevention for the general community and demonstrated an increase in knowledge change over the short-term. Based on our pre-test observations, we recommend that public health messages should continue to raise awareness regarding the asymptomatic nature of osteoporosis, and provide greater clarity about the types of physical activity that target bone. It is imperative that the recommended guidelines for osteoporosis prevention are translated into accessible messages for the public.

## Abbreviations

BMD: Bone mineral density
BSD: Barwon statistical division
GOS: Geelong osteoporosis study
OA: Osteoporosis Australia
